# Trends in diet-related greenhouse gas emissions and water footprint in Türkiye: Insights from the Türkiye Nutrition and Health Survey 2010 and 2017

**DOI:** 10.1017/S0007114525106144

**Published:** 2026-02-28

**Authors:** Azad Ilhan, Neslişah Rakıcıoğlu

**Affiliations:** 1 Faculty of Health Sciences, Department of Nutrition and Dietetics, Van Yüzüncü Yıl University, Van, Türkiye; 2 Faculty of Health Sciences, Department of Nutrition and Dietetics, Hacettepe University, Ankara, Türkiye

**Keywords:** Greenhouse gas emissions, Water footprint, Sustainability, Sustainable nutrition

## Abstract

Sustainable diets can improve environmental health by supporting food security and promoting healthy living for future generations. This study aimed to assess changes over time in the consumption of foods within the national diet and diet-related environmental indicators, specifically greenhouse gas emissions (GHGE) and water footprint (WF). Individual food consumption was assessed using 24-hour dietary recalls from the Türkiye Nutrition and Health Surveys (TNHS) conducted in 2010 and 2017. GHGE and WF were calculated based on these dietary data. According to the TNHS 2010 and 2017, GHGE increased by 16·1 %, total WF by 17 %, green WF by 19·3 %, blue WF by 9·4 % and grey WF by 10·9 % (*p* < 0·001). During the same period, the consumption of red meats (by 72 %), eggs (by 42·5 %) and fats (by 53·6 %) increased significantly (*p* < 0·001). Conversely, the most notable decrease in consumption was observed for fresh vegetables and fruits, which declined by 17·5 % and 6·9 %, respectively (*p* < 0·001). In 2010 and 2017, red meats (GHGE: +29·8 %; total WF: +23·6 %) and fats (GHGE: +14·3 %; total WF: +13·6 %) were the foods that increased their contribution to GHGE and total WF the most. Although the GHGE and total WF values of Türkiye’s national diet remain below the global average, both indicators increased in 2017 compared to 2010. Despite the rising consumption of animal-based foods in recent years, the predominance of cereals in the national diet has played a key role in keeping GHGE and total WF below the global average.

Increasing food consumption due to the rapid increase in the world population, limited energy resources and inefficient use of existing resources brings the concept of sustainability to the agenda. The food production, supply and consumption chains significantly impact the natural environment, and ensuring healthy and sustainable nutrition for the growing world population is becoming increasingly challenging^(1)^. Environmental pollution, climate change, environmental stress, biodiversity loss and increases in nutrition-related diseases pose challenges for society and the food system. The global population is expected to reach 10 billion by 2050^(2)^, making accessing adequate and healthy food difficult. Concerns are raised about the effects of the quantity and quality of food consumed on human health. In particular, obesity, diabetes, cardiovascular diseases and other chronic diseases associated with overnutrition and poor food quality are on the rise worldwide^(3)^. Consequently, sustainable and higher-quality foods must be produced for a healthy planet and a growing global population^(2,4)^.

Sustainable diets, defined as diets with low environmental impacts that contribute to food security and healthy living for future generations, have the opportunity to improve environmental health. Some foods (e.g., red meat and animal products) contribute more to diet-related environmental factors than other foods, and diets containing high amounts of these foods have higher negative environmental impacts than plant-based diets^(5)^. Sustainable food consumption significantly improves global nutrition/health standards while reducing human environmental footprints. Food systems need to be transformed to achieve the United Nations 2030 Sustainable Development Goals, which include safe and nutritious food, improved health and better management of agricultural systems and natural resources^(6)^. Calculating the impact of current dietary patterns on environmental sustainability is critical for more sustainable food systems.

The effect of the national diet on environmental factors was assessed in Türkiye^(7)^. This study aimed to determine the change in food consumption within the national diet over the years. It also assessed how these changes contributed to greenhouse gas emissions (GHGE) and water footprint (WF). This is the first study to assess changes in diet-related environmental factors (GHGE and WF) nationally in Türkiye over the years.

## Materials and methods

This descriptive study assesses the national diet’s GHGE and WF using data from the 2010 and 2017 Türkiye Nutrition and Health Survey (TNHS). The sample size was 9012 individuals (3390 males, 5622 females) for TNHS 2010 and 12318 individuals (5516 males, 6802 females) for TNHS 2017. The TNHS 2010 and 2017, conducted by the Turkish Ministry of Health, have representative sample sizes for Türkiye. The appropriateness of the study for the use of TNHS data was approved by the decision of the Hacettepe University Non-Interventional Clinical Research Ethics Committee with the registration number GO 19/1177. In addition, the necessary permission was obtained from the Ministry of Health (E-49654233-604.02).

### Dietary intake assessment

Individual food consumption was assessed by 24-h recall in the TNHS 2010 and 2017. In TNHS 2010, the 24-h recall was taken for one day^(8)^, while in TNHS 2017, it was taken twice with an interval of 2 weeks (10–14 d) in line with the recommendation of the European Food Safety Authority^(9)^. On the first day, the interview was performed face-to-face; on the second day, the individuals were scheduled for face-to-face or telephone interviews^(10)^. The ‘Food and Nutrition Photo Catalog: Measurements and Quantities’ was used to determine the quantities of food and beverages consumed^(11)^. ‘Standardized Recipes’ were used to determine the amounts of foods included in the meals consumed^(12)^. The diet’s GHGE and WF were calculated based on the gram amounts of the foods consumed.

### Greenhouse gas emissions

The diet-related GHGE calculation was made using the database developed by Heller et al.^(13)^. Again, the carbon footprint value of the consumers was determined by using the CO_2_ equivalent (CO_2_eq) values per kg specified in the database for each food through the current consumption amount and the type of food consumed. In calculating GHGE, the values of similar foods were used for foods not available in the databases.

### Water footprint

The diet-related WF was calculated using the database developed by Hoekstra et al.^(14,15)^. The WF of individuals was determined by using the WF value per ton specified in the database for each food based on the amount of consumption and type of food consumed. Again, blue, green and grey WF were calculated for each individual based on the foods consumed daily. The individual assessments’ results determined the entire sample’s total WF and the percentage distributions according to blue, green and grey WF. In calculating the WF, the values of similar foods were used for foods unavailable in the databases.

### Statistical analysis

The SPSS23 package programme was used for statistical analysis. The suitability of the data for normal distribution was examined with histogram and detrended plot graphs, kurtosis and skewness/asymmetry coefficients, coefficient of variation and Kolmogorov–Simirnov or Shapiro–Wilks tests considering the number of data. An independent samples *t*-test was used to evaluate the statistical significance of the difference between the means of two normally distributed variables, and the Mann–Whitney *U* test, which is the nonparametric equivalent of the test, was used to evaluate the differences between the means of non-normally distributed variables.

## Results

Table 1 shows the changes in GHGE and WF of the national diet in Türkiye over the years. According to TNHS data, GHGE increased by 16·1 %, total WF by 17.6 %, green WF by 19·3 %, blue WF by 9·4 % and grey WF by 10·9 % in 2017 compared to 2010 (*p* < 0·001).

**Table 1.** The greenhouse gas emissions and water footprint of the national diet in Türkiye by year

Diet-related environmental factors	TNHS 2010 (*n* 9012)			TNHS 2017 (*n* 12 318)			Change (%)	*p*
	Mean	sd	%	Mean	sd	%		
Greenhouse gas emissions (kg CO_2_eq/person/d)	2·73	2·04		3·17	1·98		+16·1	<0·001
Total water footprint (L/person/d)	2386·6	1320·1		2805·5	1324·3		+17·6	<0·001
Green water footprint (L/person/d)	1933·7	1144·9	81·0 %	2307·2	1145·3	82·3 %	+19·3	<0·001
Blue water footprint (L/person/d)	262·6	131·6	11·0 %	287·2	132·1	10·2 %	+9·4	<0·001
Grey water footprint (L/person/d)	190·3	86·2	8·0 %	211·1	119·2	7·5 %	+10·9	<0·001

*Abbreviations*: TNHS, Türkiye Nutrition and Health Survey.

Mann–Whitney *U* test (*p* < 0.05).

%, *n*: Greenhouse gas emissions and water footprint contribution percentage.

According to the TNHS 2010 and 2017 conducted at the national level, daily red meat consumption was 25·0 g/d and 43·0 g/d, egg consumption was 22·6 g/d and 32·2 g/d, respectively, and fat consumption was 8·4 g/d and 12·9 g/d, respectively. In 2010 and 2017, consumption of red meats (72 %), eggs (42·5 %) and fats (53·6 %) increased significantly (*p* < 0·001). However, the most significant decreases were observed for fresh vegetables (TNHS 2010:305·4 g/d; TNHS 2017:251·9 g/d) and fruits (TNHS 2010:170·6 g/d; TNHS 2017:158·9 g/d) (–17·5 % and –6·9 %, respectively) (*p* < 0·001) (Table 2).

**Table 2.** Daily consumption of foods and their contribution to greenhouse gas emissions and total water footprint

Food groups	TNHS 2010			TNHS 2017			Change (%)			*p* ^1^	*p* ^2^	*p* ^3^
	Consumption (g/d)	GHGE contribution (%)	Total WF contribution (%)	Consumption (g/d)	GHGE contribution (%)	Total WF contribution (%)	Food consumption	GHGE	Total WF			
Red meat	25·0	30·9	19·1	43·0	40·1	23·6	+72·0	+29·8	+23·6	<0·001	<0·001	<0·001
Poultry	26·3	4·6	5·2	25·1	2·9	3·6	−4·6	−37·0	−30·8	<0·001	<0·001	<0·001
Egg	22·6	3·2	5·5	32·2	3·4	5·9	+42·5	+6·2	+7·3	<0·001	<0·001	<0·001
Milk and dairy products	187·6	23·5	18·8	190·4	16·9	14·1	+1·5	−28·1	−25·0	<0·001	<0·001	<0·001
Cereals	257·9	4·4	23·3	247·8	3·2	16·9	−3·9	−27·3	−27·5	<0·001	<0·001	<0·001
Vegetables	305·4	7·7	2·9	251·9	4·4	1·8	−17·5	−42·9	−37·9	<0·001	<0·001	<0·001
Fruits	170·6	7·1	3·8	158·9	1·8	2·7	−6·9	−74·6	−28·9	<0·001	<0·001	<0·001
Sugar^*^	36·7	1·9	3·9	39·1	1·7	3·4	+6·5	−10·5	−12·8	<0·001	<0·001	<0·001
Fats	8·4	0·7	2·2	12·9	0·8	2·5	+53·6	+14·3	+13·6	<0·001	<0·001	<0·001
Oils	20·1	2·2	5·2	21·9	1·6	4·3	+9·0	−27·3	−17·3	<0·001	<0·001	<0·001
Black tea^†^	480·2	10·0	3·2	490·1	7·7	2·5	+2·1	−23·0	−21·9	<0·001	<0·001	<0·001

**Abbreviations:** TNHS, Türkiye Nutrition and Health Survey; GHGE, Greenhouse gas emissions; WF, Water footprint.

Independent samples t test (*p* < 0.05). *p* ^1^: (Consumption: TNHS 2010–2017), *p* ^2^: (Greenhouse gas emissions contribution: TNHS 2010–2017), *p* ^3^: (Total water footprint: TNHS 2010–2017).

* Sugar, confectionery, chocolates, jams, honey, wafers, biscuits, crackers, cakes and pastries.

† mL/d.

**Figure 1.**
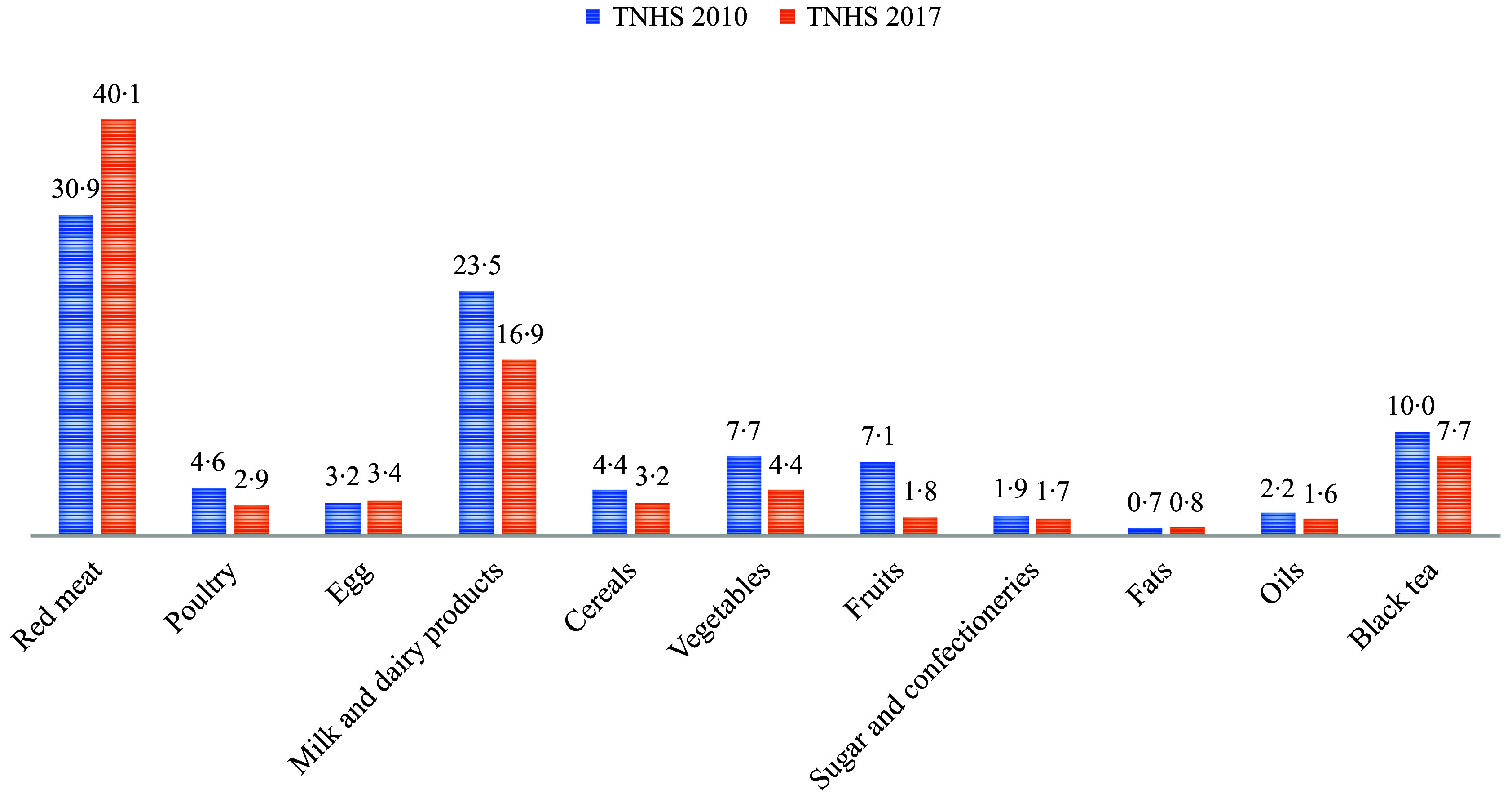
Contribution of foods to greenhouse gas emissions by year (%).

**Figure 2.**
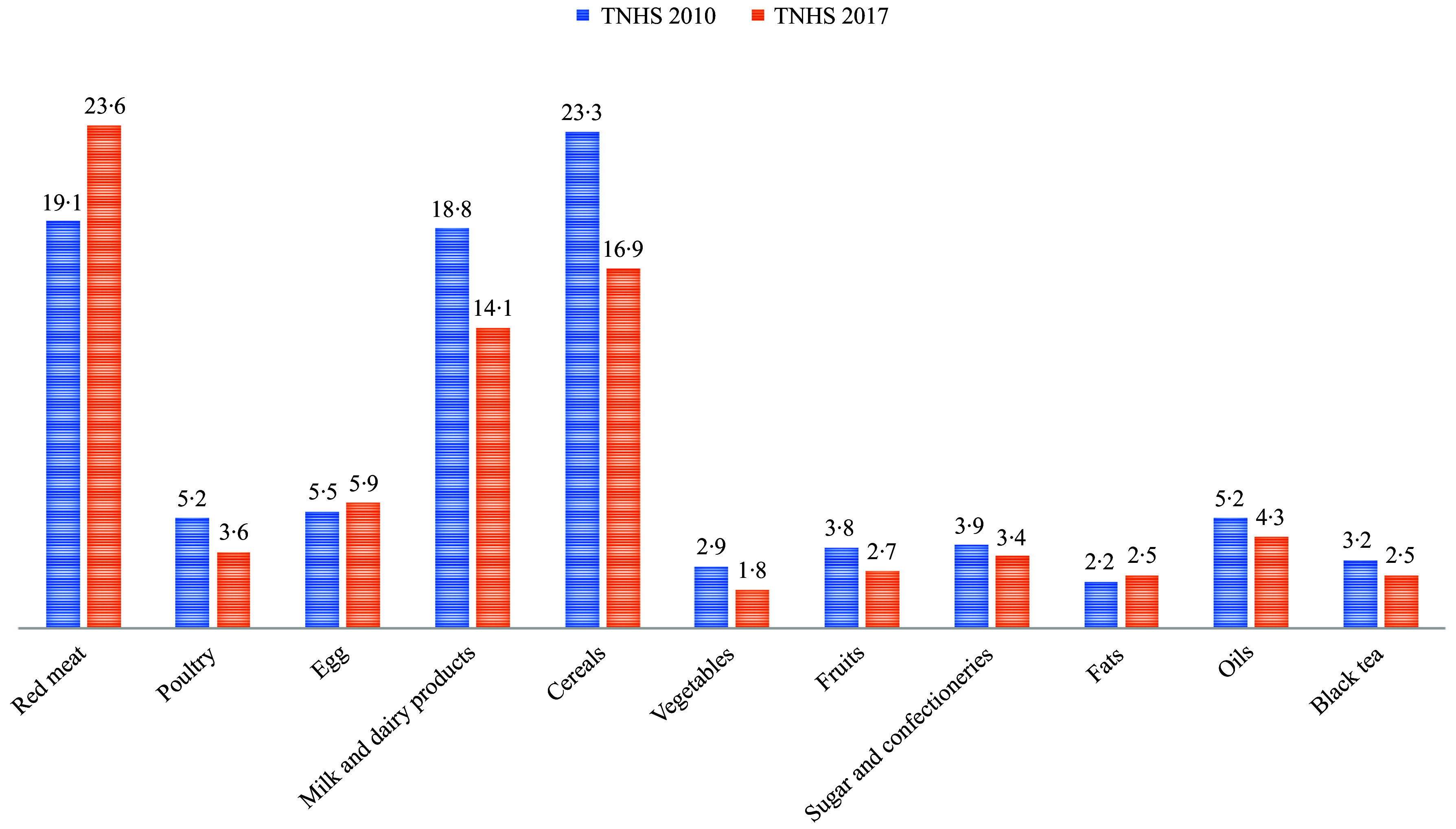
Contribution of foods to total water footprint by year (%).

Table 2, Figures 1 and 2 show the daily consumption amounts of foods, their contribution to GHGE, total WF and the changes in these values according to the TNHS 2010 and 2017. In TNHS 2010, red meats (30·9 %) and dairy products (23·5 %) made the highest contribution to GHGE, while fats (0·7 %) and sugar and confectionery (1·9 %) made the lowest contribution. In TNHS 2017, red meats (40·1 %) and dairy products (16·9 %) made the highest contribution to GHGE, while fats (0·8 %) and oils (1·6 %) made the lowest contribution (*p* < 0·001) (Figure 1). According to TNHS 2010 data, cereals (23·3 %) and red meats (19·1 %) made the highest contribution to the total WF, while fats (2·2 %) and vegetables (2·9 %) made the lowest contribution. In TNHS 2017, red meats (23·6 %) and cereals (16·9 %) made the highest contribution to the WF, while vegetables (1·8 %), fats (2·5 %) and black tea (2·5 %) made the lowest contribution (*p* < 0·001) (Figure 2).

In 2010 and 2017, red meats (GHGE: +29·8 %; total WF: +23·6 %) and fats (GHGE: +14·3 %; total WF: +13·6 %) were the foods that increased their contribution to GHGE and total WF the most. Fruits and vegetables (–74·6 % and –42·9 %, respectively) were found to have a decreasing contribution to GHGE. Vegetables and poultry were the foods with the greatest decreases in their contribution to the total water footprint, declining by 37·9 % and 30·8 %, respectively (*p* < 0·001) (Table 2).

## Discussion

The change in the environmental impact of the national diet consumed in Türkiye in 2010 and 2017 was assessed by calculating the contribution of foods to GHGE and WF based on their daily consumption amounts. This is the first study to assess the change in the environmental impact of the national diet in Türkiye through individual food consumption.

Although the GHGE of the Turkish national diet increased in 2017 compared to 2010 (2·73 ± 2·04 kg CO_2_eq/d and 3·17 ± 1·98 kg CO_2_eq/d), they are below the world average compared with other country studies where diet-related environmental factors were calculated with similar methods (Table 1). GHGE values are 3·01 kg CO_2_eq/d in Spain^(16)^, 3·4 kg CO_2_eq/d in Australia^(17)^, 4·51 kg CO_2_eq/d in Vietnam^(18)^, 4·67 kg CO_2_eq/d in Chile^(19)^, 5·0 kg CO_2_eq/d in the Netherlands^(20)^, 5·2 kg CO_2_eq/d in Italy^(21)^ and 5·48 kg CO_2_eq/d in Argentina^(22)^. In addition, the total WF of the national diet in Türkiye increased significantly in 2017 compared to 2010 (2386·6 ± 1320·1 L/person/d and 2805·5 ± 1324·3 L/person/d, respectively) (Table 1). Nevertheless, the total WF of the diet is also lower than in Brazil (3478·4 L/person/d)^(23)^, in Iran (4110 L/person/d)^(24)^, in Chile (4177 L/person/d)^(19)^ and in Mexico (6619·5 L/person/d)^(25)^. Despite the increase in animal-based food consumption in Türkiye over the years, the fact that the national diet is based on cereals has contributed to GHGE and total WF values below the world average. In Türkiye, the population’s staple foods are bread (179·8 g/d) and other cereal products (73·6 g/d)^(10)^.

The results of food consumption surveys show that red meat in the diet worldwide is the most significant contributor to GHGE and total WF^(13,23,25,26)^. Depending on the country where the research was conducted, dairy products^(10,25,26)^, corn products^(27)^, rice or legumes^(23)^ may be the second most important contributors to GHGE. According to TNHS 2010 and 2017 data, red meat and dairy products are Türkiye’s first and second most significant contributors to GHGE. While cereals and red meats were the first and second most significant contributors to the total WF in 2010, red meats and cereals were the first and second most significant contributors to the total WF in 2017. This change was driven by an increase in the consumption of red meats (72 %) and a decrease in the consumption of cereals (−3·9 %) in 2017 compared to 2010 in Türkiye (Figures 1 and 2).

Studies on the assessment of the environmental impacts of diet have primarily focused on the contributions of red meat, dairy products and cereals^(13–15)^. Unlike other countries, black tea is Türkiye’s third most significant contributor to GHGE (Table 2, Figure 1). Black tea is a traditional Turkish beverage. Daily black tea consumption was 480·2 mL in TNHS 2010 and 490·1 mL in TNHS 2017. Black tea consumption contributed 10·0 % and 7·7 % to GHGE and 3·2 % and 2·5 % to total WF for TNHS 2010 and 2017, respectively (Table 2). Considering the high prevalence of anaemia in Türkiye^(10)^, consuming black tea away from meals may be recommended for anemia, and drinking light tea may be recommended to reduce the effect of diet-related environmental factors.

Consumption of red meat and other animal-based foods contributes to unhealthy diets by increasing saturated fat and salt intake^(28)^. Furthermore, many studies have shown that diets rich in animal-based foods have negative environmental impacts with high GHGE and water and land use^(29,30)^. Therefore, reducing the consumption of animal-based foods has been proposed as the primary method to reduce environmental impacts. Guy et al.^(31)^ researched the impact of twelve different dietary patterns on health, environment and cost. They showed that dietary patterns in which meat consumption was reduced and/or meat was replaced with legumes or eggs were the most efficient regarding health, environment and cost. In Türkiye, per capita red meat consumption increased approximately 2·3-fold between 1961 and 2019^(32)^. In TNHS 2017, it was also found that red meat consumption increased by 72 % compared to TNHS 2010, and this increase increased red meat’s contribution to GHGE and total WF by 29·8 % and 23·6 %, respectively (Table 2). The increasing consumption trend of animal-based proteins such as red meat, chicken and eggs in Turkish cuisine can be effective in terms of sustainability^(10)^. Considering the high prevalence of anemia in Türkiye^(10)^, avoiding or reducing animal food consumption may also contribute to micronutrient deficiencies such as iron, zinc, calcium and vitamin B_12_^(33,34)^. Therefore, a holistic approach is required during assessment.

In a study conducted in Türkiye, GHGE was estimated as 4·12 kg CO_2_eq/d and WF as 4442 L/d according to the recommendations of the 2015-Türkiye Dietary Guidelines^(35)^. In this study, the change in the environmental impacts of the national diet in Türkiye was assessed based on actual individual food consumption records. Compared to the TNHS 2017 results, the GHGE and total WF calculated based on individual consumption recommendations were estimated to be about 30 % and 58 % higher, respectively. This difference in GHGE and WF values demonstrates the importance of accurately assessing the environmental impacts of a country’s national diet based on actual individual food consumption records rather than recommendations.

There is growing interest in changing the types of food consumed to improve sustainable nutrition and health. Many researchers are trying to replace protein-rich animal products such as meat, fish, eggs and milk with alternative protein sources produced by plants, edible insects, microbial fermentation and cell cultures^(36)^. This change in dietary habits can significantly reduce GHGE, pollution, biodiversity loss and land and water use^(37)^. However, the impact of the 2017 dietary profile in Türkiye on environmental factors suggests that there is no need for a radical change at this time. To prevent the negative environmental impacts of possible increases in animal-based food consumption in the future, it would be helpful to inform the public about the environmental impacts of diet and organise nutrition education programmes to explain the possible effects of dietary changes on environmental health.

The study’s strength is that it uses the 2010 and 2017 TNHS data, which are representative of Türkiye, and assesses changes in the environmental factors of the national diet over the years through individual food consumption. However, this study has several limitations. Since no national nutrition survey was conducted between 2010 and 2017 or after 2017, it has become impossible to assess changes over shorter periods. This gap limits the manuscript’s relevance to current policymaking, especially given dietary changes post-COVID-19 and economic crises. Second, 24-h recalls were used to assess diet-related environmental factors. The main limitation of the 24-h recall is its reliance on respondents’ memory, both to identify the foods and beverages consumed and to estimate their quantities. Another important limitation of food consumption records is under- or over-reporting of consumption. Third, the databases used to assess diet-related GHGE and WF have limitations. There is no database specific to Türkiye for assessing diet-related GHGE. Fertilisers, pesticides, irrigation systems and energy types used in agricultural practices and food production vary from country to country. Failure to account for these differences can lead to overestimating or underestimating diet-related environmental indicators such as GHGE and WF. In addition, limited information on the location of irrigated areas in countries, the country’s need for irrigation and the lack of detailed information on country-specific data are limitations of GHGE and WF calculation databases.

### Conclusion

Although Türkiye’s national diet’s GHGE and total WF values are below the world average, they increased in 2017 compared to 2010. Traditional dietary recommendations should be made based on each region’s climate, natural resources and cultural structure to promote a healthy and sustainable diet. After 2017, following significant events such as the COVID-19 pandemic and the economic crisis all over the world, the fact that a new national nutrition survey was not conducted in Türkiye for eight years until 2025 makes it difficult to understand the current situation and make recommendations. Organising nutrition education that emphasises the possible effects of changes in society’s diet on environmental health and foods that increase GHGE and WF will inform the public and raise awareness about the environmental impacts of diet. This initiative will contribute to promoting sustainable nutrition.

## References

[1] Serra-Majem L, Tomaino L, Dernini S, et al. (2020) Updating the Mediterranean diet pyramid towards sustainability: focus on environmental concerns. Int J Environ Res Public Health 17, 8758. 33255721 10.3390/ijerph17238758 PMC7728084

[2] Chapman J, Power A, Netzel ME, et al. (2022) Challenges and opportunities of the fourth revolution: a brief insight into the future of food. Crit Rev Food Sci Nutr 62, 2845–2853. 33401934 10.1080/10408398.2020.1863328

[3] Verma S & Hussain ME (2017) Obesity and diabetes: an update. Diabetes Metab Syndr Clin Res Rev 11, 73–79. 10.1016/j.dsx.2016.06.017 27353549

[4] Liu F, Li M, Wang Q, et al. (2023) Future foods: alternative proteins, food architecture, sustainable packaging, and precision nutrition. Crit Rev Food Sci Nutr 63, 6423–6444. 35213241 10.1080/10408398.2022.2033683

[5] Whittall B, Warwick S, Guy D, et al. (2023) Public understanding of sustainable diets and changes towards sustainability: a qualitative study in a UK population sample. Appetite 181, 106388. 36414148 10.1016/j.appet.2022.106388

[6] Conrad Z, Drewnowski A, Belury MA, et al. (2023) Greenhouse gas emissions, cost, and diet quality of specific diet patterns in the United States. Am J Clin Nutr 117, 1186–1194. 37075848 10.1016/j.ajcnut.2023.04.018

[7] Ilhan A, Yenicag R, Yalcin Pehlivan E, et al. (2023) Greenhouse gas emission and water footprint of the national diet in Turkey: results from Turkey Nutrition and Health Survey 2017. Sustainability 15, 9768.

[8] Repuclic of Türkiye Ministry of Health (2010) Turkey Nutrition and Health Survey – 2010. Ankara: Publications of the General Directorate of Health Research.

[9] European Food Safety Authority (2014) Guidance on the EU menu methodology. EFSA J 12, 3944.

[10] Repuclic of Türkiye Ministry of Health (2019) Turkey Nutrition and Health Survey – 2017. Ankara: Tiraj Printing and Publishing Industry Trade Ltd.

[11] Rakıcıoğlu N, Tek N, Ayaz A, Pekcan A (2010) Food and Nutrition Photo Catalog: Measurements and Quantities. Ankara: Ata Offset Printing.

[12] Merdol TK (2003) Standardized Recipes. Ankara: Hatipoğlu Publishing House.

[13] Heller MC, Willits-Smith A, Meyer R, et al. (2018) Greenhouse gas emissions and energy use associated with production of individual self-selected US diets. Environ Res Lett 13, 044004. 29853988 10.1088/1748-9326/aab0ac PMC5964346

[14] Mekonnen MM & Hoekstra AY (2012) A global assessment of the water footprint of farm animal products. Ecosystems 15, 401–415.

[15] Mekonnen MM & Hoekstra AY (2011) The green, blue and grey water footprint of crops and derived crop products. Hydrol Earth Syst Sci 15, 1577–1600.

[16] González CA, Bonet C, de Pablo M, et al. (2021) Greenhouse gases emissions from the diet and risk of death and chronic diseases in the EPIC-Spain cohort. Eur J Public Health 31, 130–135. 33001211 10.1093/eurpub/ckaa167

[17] Ridoutt B, Baird D & Hendrie GA (2021) Diets within environmental limits: the climate impact of current and recommended Australian diets. Nutrients 13, 1122. 33805454 10.3390/nu13041122 PMC8065846

[18] Nguyen SD, Biesbroek S, Le TD, et al. (2023) Environmental impact and nutrient adequacy of derived dietary patterns in Vietnam. Front Nutr 10, 986241. 37485385 10.3389/fnut.2023.986241 PMC10358330

[19] Gormaz T, Cortés S, Tiboni-Oschilewski O, et al. (2022) The Chilean diet: is it sustainable? Nutrients 14, 3103. 35956278 10.3390/nu14153103 PMC9370802

[20] Vellinga RE, van de Kamp M, Toxopeus IB, et al. (2019) Greenhouse gas emissions and blue water use of Dutch diets and its association with health. Sustainability 11, 6027.

[21] Mertens E, Kuijsten A, van Zanten HH, et al. (2019) Dietary choices and environmental impact in four European countries. J Cleaner Prod 237, 117827.

[22] Arrieta EM & Gonzalez AD (2018) Impact of current, National Dietary Guidelines and alternative diets on greenhouse gas emissions in Argentina. Food Policy 79, 58–66.

[23] Travassos GF, da Cunha DA & Coelho AB (2020) The environmental impact of Brazilian adults’ diet. J Cleaner Prod 272, 122622.

[24] Sobhani SR, Rezazadeh A, Omidvar N, et al. (2019) Healthy diet: a step toward a sustainable diet by reducing water footprint. J Sci Food Agric 99, 3769–3775. 30637755 10.1002/jsfa.9591

[25] Lares-Michel M, Housni FE, Aguilera Cervantes VG, et al. (2022) The water footprint and nutritional implications of diet change in Mexico: a principal component analysis. Eur J Nutr 61, 3201–3226. 35438358 10.1007/s00394-022-02878-z

[26] Auclair O & Burgos SA (2021) Carbon footprint of Canadian self-selected diets: comparing intake of foods, nutrients, and diet quality between low-and high-greenhouse gas emission diets. J Cleaner Prod 316, 128245.

[27] López-Olmedo N, Stern D, Bakhtsiyarava M, et al. (2022) Greenhouse gas emissions associated with the Mexican diet: identifying social groups with the largest carbon footprint. Front Nutr 9, 791767. 35433790 10.3389/fnut.2022.791767 PMC9010525

[28] Castañé S & Antón A (2017) Assessment of the nutritional quality and environmental impact of two food diets: a Mediterranean and a vegan diet. J Cleaner Prod 167, 929–937.

[29] Alexander P, Brown C, Arneth A, et al. (2016) Human appropriation of land for food: the role of diet. Global Environ Change 41, 88–98.

[30] Behrens P, Kiefte-de Jong JC, Bosker T, et al. (2017) Evaluating the environmental impacts of dietary recommendations. Proc Natl Acad Sci U S A 114, 13412–13417. 29203655 10.1073/pnas.1711889114 PMC5754780

[31] Guy DJ, Bray J & Appleton KM (2024) Select dietary changes towards sustainability: impacts on dietary profiles, environmental footprint, and cost. Appetite 194, 107194. 38154573 10.1016/j.appet.2023.107194

[32] Our World in Data (2020) Daily meat consumption per person. https://ourworldindata.org/grapher/daily-meat-consumption-per-person (accessed April 2025).

[33] Derbyshire E (2017) Associations between red meat intakes and the micronutrient intake and status of UK females: a secondary analysis of the UK National Diet and Nutrition Survey. Nutrients 9, 768. 28718824 10.3390/nu9070768 PMC5537882

[34] Arslan S, Aydın A, Gerboğa R, et al. (2024) Innovative approaches to integrating plant-based nutrition in clinical care: a path to better patient outcomes. Clin Sci Nutr 6, 175–190.

[35] Kemaloglu M, Oner N & Soylu M (2023) Environmental impacts and diet quality of popular diet models compared to Turkey’s national nutrition guidelines. Nutr Diet 80, 183–191. 36372900 10.1111/1747-0080.12785

[36] McClements DJ (2020) Future foods: a manifesto for research priorities in structural design of foods. Food Funct 11, 1933–1945. 32141468 10.1039/c9fo02076d

[37] Parodi A, Leip A, De Boer I, et al. (2018) The potential of future foods for sustainable and healthy diets. Nat Sustain 1, 782–789.

